# Editorial: Improving Mental Health for Immigrant Populations

**DOI:** 10.3389/fpsyt.2021.785137

**Published:** 2021-10-28

**Authors:** Margarita Alegría, Tiffany Yip, Amy Marks, Linda Juang, Lauren Cohen, Fernando Cuervo-Torello

**Affiliations:** ^1^Disparities Research Unit, Department of Medicine, Massachusetts General Hospital, Boston, MA, United States; ^2^Department of Medicine, Harvard Medical School, Boston, MA, United States; ^3^Department of Psychiatry, Harvard Medical School, Boston, MA, United States; ^4^Department of Psychology, Fordham University, New York, NY, United States; ^5^Department of Psychology, Suffolk University, Boston, MA, United States; ^6^Diversity in Education and Development Group, University of Potsdam, Potsdam, Germany

**Keywords:** immigrants, refugee, migrant, mental health, stress, psychological distress, children, adults

## Introduction

According to the World Migration Report of 2020, there are 281 million international migrants, three times more than five decades ago (1). Despite extensive research on the mental health challenges and outcomes within the global immigrant community (2) the field continues to confront obstacles in providing timely and evidence-based mental health care. This Research Topic of Frontiers, “*Improving Mental Health for Immigrant Populations*,” covers a wide range of topics while providing a deep dive into the differential risks of what immigrants (refugees and second and third generation children of immigrants) confront. It examines the macro and micro factors that contribute to immigrants' mental health. The Research Topic's contributions also describe new interventions or programs that offer evidence-based care for improving immigrants' mental health and identifies areas where more information is needed.

## Differential Risk of Immigrant Populations

There are significant differences between age groups and the development of mental health disorders. Cano and Takeuchi found that immigrants who arrived in the US between the ages of 0–11 were significantly more likely to meet criteria for a substance use disorder or a co-occurring disorder than those arriving as adults. Also of note, the prevalence of types of childhood adversity experienced by immigrants differed from those observed in the general US adult population. Findings from this study underscore the importance of early intervention with immigrant youth residing in the United States, and of the urgent need for integrated substance use treatment and mental health services for immigrants in the United States.

Not only are there risks associated with immigration for children but also for the mothers who leave their children behind. The review by Pineros-Leano et al. reported mixed findings of transnational mothers facing emotional difficulties when separating from their children. Differences in emotional difficulties were varied, ranging from being sad and hopeless to experiencing depression symptoms. Coping mechanisms, such as creating a reliable line of communication between mothers and children can aid in maintaining closeness and connectedness, and potentially serve to buffer the effects of transnational migration for immigrants' mental health. Understanding protective factors for first-generation immigrants and risk factors for subsequent generations can provide valuable insight on how to improve transgenerational mental health for immigrant populations.

Suárez-Orozco and López Hernández investigate the differences in mental health risk of college-aged immigrants based on their documentation status. The researchers posited that differences in undocumented status could influence mental well-being through anxiety. Factors such as time of arrival, proximity to the host country, and documentation status should be considered to better understand differences in the presentation of mental health disorders among immigrant populations.

## Addressing Immigrant Mental Health at Both the Macro and Micro Level

Addressing immigrant mental health must be done on both macro (structural/systems) and micro (personal/interpersonal) levels. Research has suggested that culturally-tailored and trauma-informed mental health training for diverse stakeholders can reduce negative effects associated with immigration and improve the adaptation experience in the settlement country (3, 4). One study in the Research Topic examined the psychological effects of asylum interviews for asylum-seekers, which has a detrimental psychological impact on refugees. Vukčević Marković et al. observe that training programs targeted at practitioners and decision-makers can lead to a process of asylum determination that is more sensitive to the asylum-seekers' mental health. They underline how refugees' mental health is influenced by the refugee status application process when it functions as an additional source of stress for traumatized refugees fleeing their home countries. This work stresses the importance for not only legal aid but psychological support for those seeking asylum in European countries.

Similarly, Goreis et al. examine the association between perceived ethnic discrimination and stress among Russian immigrants in Germany, and how greater exposure to ethnic discrimination was associated with higher levels of stress. Their findings accentuate the need to increase opportunities for social support and reduce negative coping strategies (e.g., self-blame) to counteract negative effects of ethnic discrimination for Russian immigrants.

The Research Topic also identifies policies and systems and how the actors play a role in how we perceive “the other.” Chwastek et al. find that teachers in Germany reported feeling insecure on how to best address refugee children's needs and that professional competence related to their perceptions of refugee children's behavior in school. Teachers with more negative stereotypes of refugee children and less self-efficacy also perceived them to be more difficult. This study shows the importance of pre-school teachers in cultivating effective classroom environments for newly arrived immigrant children and the need to target teacher biases that contribute to misperceptions of student behavior.

Assari et al. illustrate how systemic marginalization limits immigrant adults' capacity to benefit from resources (e.g., employment and neighborhood), thus curtailing anticipated benefits related to educational attainment. They evaluated the association between educational attainment and psychological distress, self-rated health (SRH), and chronic disease (CDs) of immigrant compared to native-born adults in the United States. Their results revealed that educational credentials are associated with lower odds of reporting psychological distress, poor self-rated health and decrease in chronic disease. However, having 16 or more years of education had a greater effect on health for native-born adults in the United States compared to immigrant adults. Assari et al.'s work underscores how we must go beyond increasing access to education to find solutions to promote equality in the benefits of educational attainment.

Also related to policy, Cratsley et al. provide a comprehensive policy and practice review on the Syrian refugee crisis, with an emphasis on forced migration and displacement and its effect on mental health. Their research shows that immigrating at a younger age is associated with a greater likelihood of developing detrimental mental health outcomes. The authors describe how the crisis has contributed to a worsening of mental health conditions and sparked new cases of PTSD due to exposure to violence and displacement. An important focus of this work is how the development of mental health conditions and level of distress can vary based on the destination country and available resources for refugees.

## Evidence-Based Care

The importance of evidence-based interventions and programs is also highlighted in this Research Topic. In Bacio et al.'s study, school was a crucial setting for implementing a multi-site program, Project Options, aimed at reducing alcohol use for Latinx youth in the United States. The intervention was successful in leading to changes in attitudes toward drinking, signaling the importance of school-based training in changing intentions and behaviors of those with more lifetime drinking experience. The study supports the Project Options intervention as a promising approach to addressing drinking behaviors among Latinx youth and the importance of adapting interventions to the school-specific cultural environment.

Physical activity can also increase the likelihood of better mental health outcomes for newly arrived refugees. Forss et al. found that refugees to Sweden who engaged in greater physical activity reported better sleep quality, lower stress, and more positive mental health and vitality. The findings support providing opportunities and dedicated spaces for newly arrived refugees to engage in physical activity. Another study by Trombka et al. detailed the testing of the Mindfulness Training for Primary Care (MTPC) program that was linguistically and culturally adapted for Portuguese-speakers in the United States. Results from their study support the effectiveness of MTPC in reducing depression and anxiety symptoms, suggesting that it is a feasible, acceptable, and culturally appropriate intervention for Portuguese immigrant populations.

This Research Topic is notable for addressing methodological challenges when assessing immigrant populations with measures that have been mainly developed for English-speaking populations in the United States. Cruz-Gonzalez et al. examine the psychometric properties of items that comprise the measures of anxiety, depression and level of functioning used in the Positive Minds Strong Bodies intervention trial administered in four languages (English, Spanish Mandarin, and Cantonese). The results of the analyses indicate that the underlying theoretical constructs were conceptualized relatively the same across the four languages. However, there were some symptoms that displayed differential item functioning, emphasizing the need to perform measurement invariance tests when examining racial and ethnic disparities in mental health research.

## Gaps in the Literature

Overall, this Research Topic provides a rich and nuanced view of the importance of evaluating different dimensions of the immigrant experience to capture mechanisms that might impact immigrants' mental health. Our Research Topic also identifies gaps in knowledge to address to have a strong knowledge base for serving our immigrant populations. First, more longitudinal research focused on the protective features of the immigrant experience in the settlement country is lacking. Second, community-based approaches that are co-designed with the immigrant population would serve to pinpoint the areas identified by immigrants as having a priority in their lives. Thirdly, more information is essential on how to intervene with the social and healthcare systems that limit the prospects of immigrants to experience good mental health in the settlement country. This is an opportunity to ensure that immigrants not only adjust well but thrive in their new homelands.

## Author Contributions

MA, TY, AM, and LJ reviewed and selected the articles for inclusion in the Research Topic. MA, LC, and FC-T contributed to the initial drafting of the manuscript. TY, AM, and LJ reviewed the drafts of the manuscript and provided feedback. All authors contributed to the manuscript revision.

## Conflict of Interest

The authors declare that the research was conducted in the absence of any commercial or financial relationships that could be construed as a potential conflict of interest.

## Publisher's Note

All claims expressed in this article are solely those of the authors and do not necessarily represent those of their affiliated organizations, or those of the publisher, the editors and the reviewers. Any product that may be evaluated in this article, or claim that may be made by its manufacturer, is not guaranteed or endorsed by the publisher.
